# Risk Factors for Fungal Prosthetic Joint Infection

**DOI:** 10.7150/jbji.40402

**Published:** 2020-03-26

**Authors:** Talha Riaz, Aaron J. Tande, Lisa L. Steed, Harry A. Demos, Cassandra D. Salgado, Douglas R. Osmon, Camelia E. Marculescu

**Affiliations:** 1 Division of Infectious Diseases, Medical University of South Carolina, Charleston, South Carolina;; 2 Department of Orthopaedics and Rehabilitation, Medical University of Charleston, Charleston, South Carolina;; 3 Division of Infectious Diseases, Mayo Clinic, Rochester, Minnesota.

**Keywords:** *Candida albicans*, case-control study, fungal PJIs

## Abstract

**Background**: Fungal prosthetic joint infections (PJIs) are rare and often associated with poor outcome; however, risk factors are not well described.

**Methods**: This was a retrospective case control study among all patients with PJIs from 2006-2016 at two major academic centers. Each fungal PJI case was matched 1:1 with a bacterial PJI control by joint (hip, knee, shoulder) and year of diagnosis. We compared demographics, comorbidities, and clinical characteristics between cases and controls using chi square/Fisher's exact or Wilcoxon rank sum test. Independent risk factors were identified with multivariable logistic regression.

**Results**: Forty-one fungal PJIs occurred over the study and 61% were due to *Candida albicans*. The hip was involved in 51.2% of cases, followed by the knee (46.3%). Compared to bacterial PJI, fungal PJI cases were more likely to have received antibiotics within the previous 3 months (70.7% vs 34%, *P*=.001), wound drainage lasting >5 days (48% vs 9%, *P*=.0002), had a lower median CRP (2.95 mg/dl vs 5.99, *P*=.013) and synovial fluid white blood cell count (13,953 cells/mm^3^ vs 33,198, *P*=.007), and a higher proportion of prior two-stage exchanges (82.9% vs 53.6%, *P*=.008). After controlling for center, prolonged wound drainage (OR, 7.3; 95% CI, 2.02-26.95) and recent antibiotics (OR, 3.4; 95% CI, 1.2-9.3) were significantly associated with fungal PJI.

**Conclusion**: In our study,* Candida albicans* was the most common species in fungal PJIs and prolonged wound drainage and recent antibiotics were independent risk factors. These clinical characteristics may help providers anticipate fungal PJI and adjust management strategies.

## Introduction

The number of patients living with implanted joint prostheses continues to increase. Many of these patients are older and require revision of the joint prosthesis due to polyethylene wear or aseptic loosening 1. Just as for other foreign devices, prosthetic joint infection (PJI) is a well-documented adverse outcome. PJI is among the main reasons for failure of joint arthroplasty 2, causing significant morbidity and mortality 3, 4. Treatment requires removal of the infected prosthesis and several weeks of intravenous antibiotics, followed by revision surgery, with an estimated cost of more than $50,000 for each episode of infection 5. The most commonly implanted joints are knee and hip prosthesis, followed by shoulder arthroplasties.

While there are consensus guidelines from the Infectious Diseases Society of America with regards to treatment of bacterial infections of prosthetic joints 1, periprosthetic infections due to fungi are rare 6. They have been historically difficult to treat as well. As the number of patients undergoing joint implantation is increasing worldwide, incidence of fungal PJIs is expected to rise 7, 8.

Among the fungi, both yeasts as well as molds have been reported to cause PJIs. Candida species account for less than 1% of all PJIs, with mold infections including Aspergillus species being reported even less frequently 9, 10.

Following implantation of a prosthetic hip and knee joint, the risk of infection is 0.5 to 1.0 percent for THAs and 0.5 to 2 percent for TKA 5, 11. There have been several proposed risk factors for a fungal PJI. Most of these risk factors are identified from small case series and from observational studies. To our knowledge, no well-designed multicenter study to identify the potential risk factors for fungal PJIs has been done. Identifying potential risk factors will allow the treating physician to identify patients at risk of acquiring fungal infection of joint prosthesis. Also, identifying higher risk patients would allow for better preoperative risk assessment and focused efforts at preventing infection. Additionally, fungal PJIs portend a therapeutic challenge due to lack of any established guidelines and have had historically poor outcomes 12-13, 31. Therefore, we sought to determine such risk factors among patients cared for at two large academic centers.

## Methods

This was a retrospective case-control study performed at two large academic centers, Center A and Center B, between January 2006 and December 2016. Cases (fungal PJIs) and controls (bacterial PJIs) were matched 1:1 ratio based on year of infection and joint location (hip, knee, or shoulder). The study was approved by the institutional review board at each study site. Center A is a large tertiary care hospital and referral center in the Midwestern United States, whereas Center B is a large regional tertiary care hospital in the Southeastern US. Both centers run robust and high volume orthopedic surgery departments. At Center A, PJI database was used to identify cases with fungal PJI. At Center B, all positive reports of all cultures ordered by orthopedic surgeons were extracted from the laboratory information system. By manual review of the culture data, cases and controls were identified.

Fungal PJI was diagnosed if two or more cultures from synovial fluid or cultures obtained from a bone biopsy, or intraoperative tissue cultures yielded the same microorganism. We excluded patients who had a mixed fungal and bacterial PJI.

We identified 41 cases of fungal PJIs (33 cases from Center A and 8 cases from Center B).

Characteristics of patients with fungal and bacterial PJIs were compared.

Demographic data included patient's age, gender and race. Comorbid conditions such as diabetes mellitus, chronic kidney disease, underlying immunosuppression, malignancy, hemodialysis dependence and exposure to antibiotics lasting longer than 3 days (systemic antibacterial medications) in the last 3 months were abstracted. We recorded clinical presentation and local wound factors such as presence of erythema, sinus tract, drainage, swelling or blister formation. Duration of symptoms was recorded. Lab data including sedimentation rate, C- reactive protein, synovial fluid leukocyte count and renal function were recorded. Finally, prosthesis related factors, such as loosening, and surgical factors that included soft tissue compromise and history of prior surgeries at the index joint including two stage exchange, were abstracted. Timeline of prior surgeries was from date of index arthroplasty.

Rheumatoid arthritis was defined as per the guidelines of the American Rheumatology Society 14. Diabetes mellitus and obesity were defined as per the national diabetes data group 15, the National Institute of Health (NIH) and the World Health Organization (WHO) 16, respectively. Any form of systemic steroid use lasting longer than two weeks prior to the diagnosis of PJI was recorded. Malignancies that occurred within the past five years (before the diagnosis of PJI) were recorded as well. Chronic renal insufficiency was defined as having a glomerular filtration rate (GFR) of less than 30 mL/min/1.73 m^2^. Wound drainage was defined as any drainage occurring longer than five days before the diagnosis of PJI.

### Statistical analysis

We compared categorical variables between fungal and bacterial PJI groups using chi square or Fisher's exact test as appropriate. Continuous variables were compared with the Wilcoxon rank sum test. All tests were two-sided. Type I error (α) was set at 0.05. Risk factors we considered statistically (*P*≤.05) and clinically significant were included in a multivariable logistic regression model in a stepwise fashion (SAS 9.4), Cary, NC). The Hosmer-Lemeshow test was performed to assess goodness-of-fit of the final model.

## Results

The median age of the patients in the study was 64.7 years. Twenty-one (51%) of the 41 case patients were female and 36 (87%) were white. The hip was involved in 21 (51.2%) cases, followed by the knee (46.3%) and shoulder (2.4%). The median duration from the time of initial joint arthroplasty and diagnosis of fungal PJI was 515 days (range 17- 5,949). The clinical characteristics are summarized in Table 1.

Median BMI was 29 kg/m^2^ (range 10-106). One patient had a prior limb amputation accounting for a rather low BMI of 10 kg/m^2^ .Twenty-one (51.2%) cases had one to three co-morbid conditions which included diabetes mellitus in eight (9.5%), chronic renal insufficiency in five (5%), underlying immunosuppression in 11 (26.8%), hemodialysis dependence among six (14%) and receipt of antibiotics within the previous 90 days in 29 (70.7%) cases. While there were no major differences in age or other comorbid conditions, having received antibiotics within the previous 90 days was noted to be statistically significant (*P*=.001).

In terms of clinical presentation, pain was present in all cases (100%), followed by swelling in 30 (73.1%), presence of a sinus tract in 21 (51%), wound drainage in 20 (48%), erythema in 12 (29.2%) and fever in five cases (12.1%).

Median CRP was lower among cases (2.95 mg/dl, range 0.30- 23.9) versus controls (5.99 mg/dl, range 0.04- 38.6, *P*=.0132). Similarly, median synovial fluid leukocyte count was 13,953 cells/mm^3^ among cases versus 33,198 cells/mm^3^ among controls in whom this data was available (*P*=.007).

We examined surgical factors including surgical site skin infection, loosening of the prosthesis, soft tissue compromise as well as history of prior operation at the index joint and history of prior two stage exchange.

In the univariable analysis, prior two-stage exchange, receipt of antibiotics within the previous 90 days, wound drainage lasting longer than five days, CRP and synovial fluid leukocyte counts were statistically significant.

In the multivariable logistic regression model, after controlling for the center, presence of wound drainage for more than five days (OR, 7.3; 95% confidence interval [CI], 2.02-26.95) and receipt of antibiotics within the previous 90 days (OR, 3.4; 95% CI, 1.2-9.3) were factors significantly associated with fungal PJI.

Tables 2 and 3 enlist the microbiological characteristics of the 41 cases and controls. While *Candida albicans* was the most common fungal pathogen, the most common bacterial pathogen in our study was *Staphylococcus epidermidis*.

## Discussion

To our knowledge, this is the first case control study aimed to evaluate risk factors for fungal PJIs. Our data suggest that there are two factors significantly associated with an increased odds of fungal PJIs when compared with bacterial PJIs; antimicrobial therapy within three months before the diagnosis of PJI was associated with a 3.4-fold increased odds of having a fungal PJI, and presence of wound drainage lasting longer than five days prior to the diagnosis of PJI was associated with a 7.3-fold increased odds of fungal PJI.

In our study, consistent with what has been reported in the literature, the most commonly identified fungus was *Candida albicans,* which was recovered in twenty-five (60.9%) cases followed by *Candida parapsilosis* (24.4%) 13, 30 (Table 2). It has been reported that *Candida albicans* makes large and complex biofilms compared to other candida species 13, 17, thus contributing to its pathogenicity. Among the patients included in the bacterial cohort, coagulase negative *Staphylococcus* was recovered in 43.9%, followed by *Staphylococcus aureus* in19.5%. This distribution of bacterial pathogens is similar to what has been reported by Berbari *et al* 3. Other studies looking at microorganisms in periprosthetic hip and knee infection also noted *S aureus* and coagulase negative *Staphylococci* to be the most frequent 18, 19.

Not surprisingly, we found that receipt of antibiotics in the previous 90 days had a significant association with acquisition of fungal PJI. Previous antibiotic use selects for candidal growth and to that extent, biofilm formation over joint prosthesis is a central process of pathogenesis 1, 20-22. Additionally, large joints such as the hip and knee are at a higher risk for developing infection due to inherently low blood flow to the cortical bone and occasional formation of a hematoma in a large dead space around the prosthetic device. Hematomas can devascularize adjacent tissues and prevent delivery of antibiotics 6.

Our study found that an association between wound drainage lasting more than 5 days and being at risk for fungal PJI. Ongoing wound drainage (especially under broad spectrum antibiotics for prior bacterial infection) allows superinfection with other pathogens (eg, candida here). One could argue that optimizing the wound environment such that we can reduce fungal or bacterial deposition into the wound could potentially decrease the risk of subsequent infection 23.

Co-morbid conditions including diabetes mellitus type II, renal disease, immunosuppression, malignancy and hemodialysis dependence have been reported to be potential risk factors for fungal PJI 10, 24. In our study, 53.6% of cases had at least one co-morbid condition. However, this level of comorbidity was observed in similar frequency in the bacterial cohort. This is not surprising, given that these risk factors are also associated with bacterial PJI. None of the patients had HIV or an immunodeficiency associated condition. In one large review study performed in the Netherlands by Kuiper *et al* 25, 164 cases of fungal PJI were reviewed from 1966 to July 2012 and 68% of the patients had one or more risk factors for fungal PJI. In another study by Azzam *et al* 10, half of the patients with fungal PJI had one or more established risk factors. A more recent study by L. Escola- Verge *et al,* nearly 80 % had an identifiable risk factor for *candida* infection 30.

As noted in the previously published studies, lab data including CRP and sedimentation rate does not help discriminate fungal from bacterial PJI 26. However, in our study, we observed that the median CRP and sedimentation rates were more often lower in fungal PJIs compared to bacterial PJIs. Additionally, we observed in the univariable analysis a relatively lower median synovial fluid leukocyte count among fungal cases (*P*=.007). The lower serum CRP levels as well as synovial fluid counts in fungal PJIs may suggest that the inflammatory response is not as robust as one would expect to see in a bacterial infection.

Referencing back to the study done by Kuiper *et al* 25, in which after an extensive search of literature, the authors identified 164 cases of fungal PJIs, half had prosthetic loosening described radiographically. This is similar to what has been reported in patients with bacterial PJIs 27. In contrast, our study noted that only 26.8% of patients had loosening in the context of fungal PJIs, but this was deemed not to have a statistically significant association.

The hallmark of our study was the relatively large number of cases (82.9%) with history of a prior two-stage exchange (*P*=.008). To our knowledge, this is the first study to establish an association between a patient's surgical history and subsequent development of fungal PJI. In a study done by Geng *et al* 28, which was a retrospective analysis of eight cases of fungal PJIs, six had undergone additional surgery on the infected joint following primary total joint arthroplasty and preceding diagnosis of fungal PJI. In a second smaller study from Spain, comprising 10 patients with fungal PJIs, nine had previous bacterial infections, received antibiotics for more than 15 days and required multiple surgeries 29. We infer that prior two-stage exchange may increase the risk of fungal infection, possibly mediated through an increased exposure to antibiotics (for previous episode of infection) leading to selection for candida species.

Strengths of this study include a strict definition of cases and controls as well as potential risk factors. We only included cases where we had demonstration of fungi in at least two samples (tissue/fluid). By excluding mixed cultures, we eliminated bias as patients with mixed cultures would be inherently a different population than those infected by a single pathogen. Given the retrospective nature of this study, there might have been some inherent limitations with regards to the recording of data. Also, certain risk factors that occur infrequently may not have been identified. 87% of the patient population was Caucasian; thus the results of our study can apply primarily to the Caucasian population and we cannot make an inference to the role of other races as risk factors due to the small number. Most of the cases (33 of 41) came from Center A, which could have potentially created selection bias, however we controlled for center in the final model. Both centers acquired and collected data in a similar way and both centers used the same software. As explained by L. Escola- Verge *et al*, searching and treating for candida intertrigo could be a reasonable measure to avoid candida PJI 30. We were not able to record how many patients had cutaneous candidiasis as that can be a potential risk factor.

## Conclusions

Our study suggests that receipt of antibiotics within the previous 90 days and wound drainage for more than five days were independent risk factors for fungal PJI. More than half of the patients had at least one risk factor predisposing to fungal PJI and recognition of these risk factors could help clinicians anticipate fungi as the cause of PJI and potentially help to alter medical and surgical treatment strategies.

## Figures and Tables

**Table 1:** Demographics and Clinical Characteristics of Cases and Controls

Variable	Fungal PJI (n-41)	Bacterial PJI (n=41)	*P* Value
**Demographics**			
Age median years (range)	64.7 (43.9-81.4)	66.7 (14.3-89.1)	NS
Female	21 (51%)	20 (48%)	NS
Race white	36 (87%)	36 (87%)	NS
**Prosthesis location**			
Hip	21 (51%)	21 (51%)	
Knee	19 (46%)	19 (46%)	
Shoulder	1 (2.4%)	1 (2.4%)	
**Joint age** median days (range)	515 (17-5,949)	1,321(37-7,595)	.057
**Host Factors**			
BMI median (range)	29 (10-106)	29 (24-61)	.35
**Comorbid conditions**			
None	19 (46.3%)	20 (48.7%)	
1-3	21 (51.2%)	20 (48.7%)	
>3	1 (2.4%)	1 (2.4%)	
Diabetes mellitus	8 (19.5%)	5 (12.1)	NS
Chronic kidney disease	5 (12.1)	7 (17%)	NS
Immunosuppression	11 (26.8)	5 (12.1)	NS
Malignancy	5 (12%)	1 (2.4%)	NS
AKI/HD	6 (14%)	2 (4.8%)	NS
Prior antibiotics (in last 3 months)	29 (70.7%)	14 (34%)	.001
**Clinical presentation and local wound factors**			
Erythema	12 (29.2)	18 (43.9)	NS
Sinus tract	21(51%)	12 (29.2%)	.07
Pain	41 (100%)	39 (95%)	NS
Fever	5 (12.1%)	8 (19.5%)	NS
Swelling/cyst	30 (73.1%)	28 (68.2%)	NS
Wound drainage (>5 days)	20 (48%)	4 (9%)	.0002
Duration of symptoms median days (range)	30.0 (1-910)	12.0 (1-1,213)	.08
**Laboratory data**			
CRP median (range) (mg/dl)^a^ (39^a^/40^b^)	2.95 (0.30-23.9)	5.99 (0.04-38.6)	.0132
Sedimentation rate median (range) (39^a^)	45.0( 6-130)	55.0 (0.9-130)	NS
Creatinine (mg/dl)	0.9 (0.5-1.7)	1.0 (0.5-9.1)	NS
Synovial fluid leukocyte (cells/mm^3^) median (range) (24^a^/26^b^)	13,953 (157-84,378)	33,198 (350-251,049)	.007
**Surgical factors**			
Surgical site infection	4 (9%)	1 (2.4%)	NS
Loosening	11 (26.8%)	9 (21.9%)	NS
Soft tissue compromise	7 (17%)	2 (4.8%)	NS
Prior operation at the index joint	36 (87.8%)	28 (68.2%)	.06
Prior two stage exchange	34 (82.9%)	22 (53.6%)	.008

Abbreviations: NS, not significant; PJI, prosthetic joint infection. ^a^ Number of cases for whom risk factor data were available. ^b^ Number of controls for whom risk factor data were available.

**Table 2:** Microbiological Findings of 41 Patients with Fungal PJIs (Cases) Seen at Center A and Center B between 2010 and 2016

Fungal pathogen	Number of patients
*Candida parapsilosis*	10
*Candida albicans*	25
*Candida guilliermondii*	1
*Candida tropicalis*	1
*Coccidiodes immitis*	1
*Candida glabrata*	2
*Bipolaris spp.*	1

Abbreviation: PJI, prosthetic joint infection.

**Table 3:** Microbiological Findings of 41 Patients With Bacterial PJIs (Controls) Seen at Center A and Center B Between 2010 and 2016

Bacterial pathogen	Number of patients
*Staphylococcus epidermidis*	18
Group B *Streptococcus*	2
Methicillin susceptible *S aureus*	6
Microbiologically indeterminate	1
*Enterococcus faecalis*	2
*Staphylococcus lugdenensis*	2
*Serratia marcescens*	1
*Cutibacterium acnes*	2
*Streptococcus viridans*	1
*Streptococcus mitis*	2
Methicillin resistant *S aureus*	2
*Micrococcus luteus*	1

Abbreviation: PJI, prosthetic joint infection. Brown, T. S. *et al.* (2018) 'Periprosthetic Joint Infection With Fungal Pathogens', *The Journal of arthroplasty*, 33(8), pp. 2605-2612.

## Abbreviations

PJI: prosthetic joint infection
THA: total hip arthroplasty
TKA: total knee arthroplasty
AKI: acute kidney injury
